# Normalization of Non-Drinking? Health, School Situation and Social Relations among Swedish Ninth Graders That Drink and Do Not Drink Alcohol

**DOI:** 10.3390/ijerph182111201

**Published:** 2021-10-25

**Authors:** Jonas Raninen, Peter Larm, Johan Svensson, Michael Livingston, Lars Sjödin, Patrik Karlsson

**Affiliations:** 1 Swedish Council for Information on Alcohol and Other Drugs (CAN), 116 64 Stockholm, Sweden; jonas.raninen@can.se (J.R.); johan.svensson@can.se (J.S.); 2 Karolinska Institutet, Department of Clinical Neuroscience, 171 77 Stockholm, Sweden; michael.livingston@curtin.edu.au (M.L.); lars.sjodin@ki.se (L.S.); 3 Unit of Social Work, School of Social Sciences, Södertörn University, 141 89 Huddinge, Sweden; 4 Centre for Alcohol Policy Research, La Trobe University, Melbourne, VIC 3086, Australia; 5 Department of Public Health Sciences, Stockholm University, 106 91 Stockholm, Sweden; peter.larm@su.se; 6 National Drug Research Institute, Curtin University, Melbourne, VIC 3004, Australia; 7 Department of Social Work, Stockholm University, 106 91 Stockholm, Sweden

**Keywords:** alcohol, adolescent, survey, Sweden, non-drinking

## Abstract

Alcohol consumption is a major contributor to the disease burden among adolescents. The adolescent alcohol abstainer is still often depicted as problematic in the research literature and in prominent theoretical frameworks. However, over the past two decades, there has been a marked trend of declining youth drinking in Sweden. The declining trend has led to a shift in the majority behaviour of youth, from drinking to non-drinking. It is plausible that this trend has also shifted the position of non-drinkers. This paper examines the position of non-drinkers in a nationally representative sample of Swedish adolescents. A survey was carried out in 2017 in 500 randomly selected schools. A total of 5549 respondents (15–16-year-olds) agreed to participate and answered the questionnaire. A minority (42.8%) had consumed alcohol during their lifetime. The results show that non-drinkers had better health and school performance when compared to drinkers. The results also showed that there were no differences in the social position between non-drinkers and drinkers. These findings are new and indicate a changed position of non-drinkers among Swedish adolescents. With non-drinking being the majority behaviour among Swedish adolescents this seems to have shifted the position of non-drinkers. There is a need for research on the long-term importance of not drinking during adolescence.

## 1. Introduction

Alcohol is a major contributor to the global burden of disease [1] and is the leading risk factor for ill-health among Swedish youth [2]. Somewhat paradoxical the adolescent abstainer is often depicted as problematic in the literature, most often because of poorer social relations [3,4,5,6].

There is a paucity of general population studies focussing on alcohol abstainers. This is most likely due to the fact that in most European countries alcohol consumption is normative with a majority of the population being alcohol consumers. In Sweden, nine out of ten adults have consumed alcohol during the past year [7]. That most research efforts are directed towards understanding the majority of behaviour is natural, especially since alcohol consumption also causes a lot of harm. It has, however, been noted in the literature that alcohol researchers tend to be problem oriented [8], which most likely has contributed to the lack of studies on those not drinking alcohol.

Drinking also used to be the norm among youth. For example, in the past year prevalence rates of alcohol consumption were between 80 and 90 per cent among Swedish ninth graders between the 1970s and 1990s [9]. Consequently, non-drinking during these decades was the exception, not the norm.

A commonly found result is that non-drinking youth, compared to drinkers, have better somatic health and perform better in school [3,10]. However, they have lower sociability in that they have fewer friends [3], are less satisfied with their relationships with friends [11,12] and have more problems finding new friends [10]. A study by Leifman and colleagues [3] also found that in addition to lower sociability the abstainers also experienced more mental health problems. Using data from a Swedish sample born in 1950 and 1951, they observed that only 6% were abstainers at the age of 18. Leifman et al. attributed these findings to non-drinkers belonging to a marginalized group, not engaging in the majority behaviour [3]. Support for this interpretation is that alcohol consumption for most people is a social activity [13] and drinking has been seen as key for unlocking access to the social arenas for youth. Previous research also describes drinking as normative and idealised among youths, and non-drinkers at risk of social isolation [14,15]. Common for all of these studies is that they analysed data from samples and time periods where drinking was the majority behaviour.

Several prominent theoretical frameworks also depict the adolescent abstainer as problematic. Both Problem Behavior Theory (PBT) and Moffitt’s theory of delinquency describe the adolescent abstainers as groups troubled by problems [16,17]. Moffitt attributes this to the fact that most adolescents take part in norm breaking behaviour to some extent, that this is normative and part of this phase in the life-course. Those not engaging in and experimenting with norm breaking behaviour are thought to be excluded from the social arenas and thus do not have the same possibilities as the majority. Similarly, Jessor’s PBT defines problem behaviour as something departing from conventional behaviour [16], i.e., it is not inherently a bad or risky behaviour. Problem behaviour according to this definition is also relative to the age or stage in life the person is in. Some behaviours can be regarded as deviant and problematic at a certain age whilst normal at other ages. Problem behaviour is therefore defined as “a departure from the regulatory norms defining appropriate behaviour for that age or stage in life” ([16], p. 30).

However, whilst such models may have been useful for the understanding of non-drinking during prior decades, there are strong indicators that they now are quite outdated. Since the turn of the millennium, youth drinking has changed radically in Sweden as elsewhere. The prevalence of drinking among 15–16-year-olds has steadily declined from 81 per cent in the year 2000 to only 39 per cent in 2018 [18]. Since 2013, when the prevalence of drinking dropped to 47 per cent, drinking is no longer the majority behaviour among Swedish ninth graders. Similar trends have been observed in most western countries [19,20,21,22]. Against this background with trends of declining drinking and increase of non-drinkers that has been so marked that it has also led to a shift in the majority behaviour, there is a need to assess what the changing social status of drinking implies for our understanding of non-drinking in adolescence.

According to the normalization theory by Parker et al. [23], when a low prevalent behaviour becomes widespread in a population previous knowledge about its correlates, based on our understanding of the phenomenon as deviant, will no longer work. The behaviour previously isolated to sub-groups and sub-cultures will have spread to the wider population and the former deviant users are then mixed up with ‘normal’ users, rendering previous results and knowledge of correlates void. Trying to understand behaviours that have become normalized using pathologizing theories will not be useful and the authors argue that we need to change our approach as the behaviour changes [24]. While the normalization theory originally concerned the use of illicit drugs, it should be equally relevant for other behaviour, such as abstaining, as well.

The aim of the present study is therefore to use a nationally representative sample of Swedish ninth graders from 2017, where the majority were non-drinkers, to compare Swedish youth that drank alcohol with those not drinking with respect to their social relations, self-rated somatic health, mental health and school situation. Besides these overall comparisons, we also compare drinkers and non-drinkers separately for boys and girls. The purpose of these comparisons is not to assess causal relations, but to examine the social markers of alcohol consumption in light of the marked changes in youth drinking that has occurred over the past two decades in Sweden.

## 2. Materials and Methods

This study used a national sample of Swedish ninth graders (15–16-year-olds) collected in 2017. A random sample of 500 schools was drawn by statistics Sweden (SCB). A probability proportional-to-size sampling design (PPS) was applied where a school’s inclusion probability was proportional to its number of ninth-grade students. The participation rate for schools was 68.6% (*n* = 343). In each of the sampled schools, one class was then selected using a PPS sampling procedure. All students in the selected class who were present on the day of the survey were then asked to participate in the study. Those agreeing to participate were asked to fill out a self-administered paper-and-pen questionnaire during school hours. The sample comprised 5549 ninth grade students and the response rate was 82.3%. The study was approved by the ethical review board of Stockholm (2017/5:2).

### 2.1. Measures

#### 2.1.1. Drinking Status

This was measured with the question “Have you ever had a drink of alcohol?”, with the response alternatives “No/Yes, during the past 30 days/Yes, during the past year/Yes, more than 12 months ago”. This was dichotomised into “No = 0” and “Yes = 1”.

#### 2.1.2. Heavy Episodic Drinking

Heavy episodic drinking (HED) was measured with the question “How often do you have six or more drinks on one occasion?”, with the response alternatives “Never/Less than monthly/Monthly/Weekly/Daily or almost daily”. This was dichotomised into “Less than monthly = 0” and “Monthly or more often = 1”.

#### 2.1.3. Psychosomatic Problems

Psychosomatic problems (PSP) were measured with five questions. “During the past six months, how often have you… had stomach aches, felt stressed, had difficulties keeping awake during class, had trouble falling asleep, had headaches?”. Response alternatives were “Every day = 5, A few times per week = 4, Once a week = 3, A few times per month = 2, More seldom or never = 1”. These were summarized into an overall PSP-scale, range 5–25 (Cronbach’s alpha 0.74). Higher values on this composite measure were thus indicative of more psychosomatic problems, either through experiencing several problems or a higher frequency of problems.

#### 2.1.4. Self-Rated Health

Self-rated somatic health (SRH) was measured with the question “If you think about your health in general, how would you say that you feel?”, with the response options “Very good = 5, Good = 4, Neither good or bad = 3, Poor = 2 and Very poor = 1”.

#### 2.1.5. Strengths and Difficulties Questionnaire

Three sub-scales from the Strengths and Difficulties Questionnaire (SDQ) were used [25]. The Swedish one-sided self-rated SDQ for 11–17-year-olds was used. The questionnaire consists of 25 items on psychological attributes with five subscales that contains five items each. Internalizing problems consist of the subscales of emotional symptoms and peer relationship problems, while externalizing problems consist of conduct problems and hyperactivity symptoms. The pro social sub-scale was also used. The question was asked; “How do the following statements correspond how you are as a person?”, with the response alternatives: “Incorrectly = 1/Partly correct = 2/Totally correct = 3”. Examples of items included are “I am constantly fidgeting or squirming/I am easily distracted, I find it difficult to concentrate” both externalizing, “I worry a lot/I would rather be alone than with people of my age” both internalizing and “I often volunteer to help others (parents, teachers, children)/I try to be nice to other people. I care about their feelings, both pro social.

#### 2.1.6. Friendship Satisfaction

Friendship satisfaction was measured with the question “How satisfied are you usually with the relationship to your friends?”, with the response alternatives “Very satisifed = 4, Satisifed = 3, Not so satisifed = 2, Not satisifed = 1 and Do not know = Missing”.

#### 2.1.7. School Performance

The respondents were asked to report their grades for the last semester in three subjects (Swedish, English and Mathematics) on a scale from A-F. These were recoded as follows; A = 20, B = 17.5, C = 15, D = 12.5, E = 10 and F = 0. This was then summarized into a scale ranging from 0–60 where higher values indicate better school performance.

#### 2.1.8. School Satisfaction

School satisfaction was measured with the question “How do you like school?”, with the response alternatives “Very good = 5, Fairly good = 4, Neither good nor bad = 3, Pretty bad = 2 and Very bad = 1”.

#### 2.1.9. Truancy

Truancy was measured with the question “Do you ever play truant?”, with the response alternatives “No = 1, Yes, a few times per semester = 2, Yes, about once a month = 3, Yes, 2–3 times per month = 4, Yes, about once a week = 5 and Yes, several times per week = 6”.

#### 2.1.10. Statistical Analysis

We included only respondents with valid answers to all variables included, resulting in a final analytical sample of 5099 (91.9% of the original sample). In the final sample, 48.9% were boys. Descriptive comparisons were made between drinkers and non-drinkers. Differences were tested using t-tests. The significance level was set at *p* < 0.05 for all analyses.

#### 2.1.11. Data Availability

The data presented in this study are available on request from the corresponding author. The data are not publicly available due to privacy and ethical reasons.

## 3. Results

Of the sample, 42.8 per cent reported ever having a drink of alcohol. Table 1 shows that non-drinkers had a lower score on the PSP-scale indicating that they are less burdened by psychosomatic problems than their drinking peers. Non-drinkers had a higher score on the pro-social sub-scale from the SDQ. There were no significant differences found for the internalizing sub-scale from the SDQ. On the externalizing sub-scale, however, the drinkers had a significantly higher score. The non-drinkers also had somewhat higher grades than the drinkers. There was a significantly higher proportion of non-drinkers that rated their somatic health better compared to drinkers. Non-drinkers also had higher school satisfaction and markedly lower rates of truancy.

The sub-group of drinkers reporting HED once a month or more often had more psychosomatic problems than the drinkers and non-drinkers, whilst there were no differences in how they rated their somatic health when compared to drinkers. The sub-group with HED also had lower grades and markedly higher rates of truancy compared to both the other groups.

From Table 2 we can see that among girls, non-drinkers rated their health better, had fewer psychosomatic problems and lower scores on both the internal and external sub-scales of the SDQ. Non-drinkers also had higher grade-point average, higher school satisfaction and lower rates of truancy. On the pro-social sub-scale of the SDQ, non-drinking girls had a somewhat higher score whilst there were no differences observed between the two groups when it comes to how satisfied they are with their friends.

For boys, the patterns were very similar to those found for girls. Non-drinking boys rated their health better, had fewer psychosomatic problems and had a lower score on the external sub-scale of the SDQ. No difference was found for the internal sub-scale of the SDQ. Non-drinkers also had higher grade-point averages, higher school satisfaction and lower rates of truancy. When it comes to social relations, non-drinking boys had a higher score on the pro-social sub-scale of the SDQ whilst they reported lower satisfaction with their friendships.

## 4. Discussion

The major aim of this study was to compare Swedish youths that drink alcohol with those that do not drink alcohol regarding health, mental health, school situation and social situation. In general, only very small differences were observed between the two groups. Where differences were found they consistently showed that non-drinking Swedish ninth graders in 2017 were better off than their drinking peers. They had fewer psychosomatic problems and fewer externalizing problems. Their school situation was also better with higher school satisfaction, higher grade point average and less truancy. This is in line with previous research showing non-drinkers to be more well-adapted and healthier [10,11,26]. Somewhat surprisingly, our results also showed that the non-drinkers had a higher score on the pro-social sub-scale from the SDQ. This indicates, contrary to previous research [3,10], that their sociability was not negatively affected by them not drinking alcohol. Our result displayed very similar patterns for boys and girls so there does not seem to be any major differences between the sexes in the importance of drinking status.

The results indicate that the marked changes in youth drinking that have occurred over the past two decades have also shifted the social marker of alcohol so that it is no longer a deviant behaviour among youth not to drink. In this ‘dry’ period it is instead the drinkers that display somewhat lower sociability and worse health. A further interpretation of our results would suggest that not drinking alcohol is no longer a deviant behaviour in this age group in Sweden according to the definition of problem behaviour by Jessor [16].

Our results echo those of two recent qualitative studies from Norway [27] and Sweden [28] that also concluded that the cultural position of alcohol has changed among adolescents. The qualitative findings suggest that alcohol has lost its importance in socializing situations between peers and that today’s adolescents do not perceive any peer pressure to drink [28] which is also supported by quantitative findings of drinking motives among Swedish youth [29].

Measham et al. studied illicit drug use among British adolescents during the 1990s when there was a sharp increase in the prevalence of use [24]. They hypothesized that when prevalence rates of illicit drug use were around 50 per cent it was no longer meaningful to try and understand illicit drug use within a pathologizing framework; illicit drug use had become normalized among adolescents in Britain, and this required a new approach to understanding illicit drug use. Even though the main focus of our study is fundamentally different, i.e., we are not studying a risk behaviour, we still think the analogy and arguments by Parker et al. are valid for our case. The major changes that have occurred in youth drinking over the past two decades have led to a shift in the majority behaviour of youth. Instead of seeing a spread of a risk behaviour we are now seeing a spread in non-drinking among youth. In our sample the majority of Swedish ninth graders were non-drinkers and the results show that there are small differences between the groups of drinkers and non-drinkers. In all aspects, non-drinkers were better-off compared to their drinking counterparts suggesting that not drinking is normalized in this group of Swedish youth. The pattern of differences and similarities between the groups of non-drinkers and drinkers also persisted when excluding a sub-group of heavy drinkers.

The trends in youth drinking are still unexplained and we do not know what is behind the decrease in drinking and the rise of non-drinking [20,22,30]. Maybe one reason for the lack of convincing explanations is that researchers are still thinking of not drinking as a minority behaviour whilst if we are to go looking for explanations for ‘normal’ youth behaviour we need other approaches, much like the case of understanding illicit drug use in Britain during the 1990s [24]. A recent study for example showed that non-drinkers were a heterogeneous group consisting of several distinct sub-groups, suggesting that we will need not one but several explanations [31].

The present study was based on cross-sectional data and all information is based on self-reports. This needs to be kept in mind when interpreting the results. The drinking status of the youth might have been wrongly reported and has only been reported on one occasion. Since data was collected at an age when many begin experimenting with alcohol several of the non-drinkers might have transitioned to drinking shortly after participating in the survey. The drinking status should thus not be interpreted as a persistent trait of the youth. The cross-sectional design of the study does not allow us any opportunity to study the direction of associations and thus we do not know if it is the drinking status of the youth that renders the differences in the other variables or if it is the other way around. Most likely several of the associations are reciprocal. Our focus in this paper, however, was on the position of non-drinkers rather than determinants or consequences of their drinking status, which should make these issues less salient. The major strength of the current study is the large nationally representative sample. The survey also had a high response rate. Data covers roughly 5–6% of all ninth graders in Sweden in 2017. Our findings should therefore be valid for the population and not subject to regional variations or particular sub-groups of Swedish youth. The large sample also provides us with sufficient statistical power so that our observations are not merely random variations between the groups.

Since the drinking status of these youth is probably not a persistent trait, future studies need to examine the prevalence of transitions to drinking and when this transition occurs. It would also be interesting to examine if non-drinkers do become marginalized at the age when the majority has started to drink or if the tolerance for non-drinkers in this ‘dry’ generation has changed permanently. There is also a paucity of studies on the importance of non-drinking in a longitudinal perspective. The results of the present study show that non-drinkers are better off in a cross-sectional perspective, but it should be studied if not drinking during adolescence also renders long term benefits.

In light of the trends reported for adolescents with sharp increases in rates of non-drinkers, we would also argue that there is a need to start researching abstinence or not drinking since this is now the majority behaviour among youth in many western countries.

## 5. Conclusions

Over the past two decades, there has been a trend of declining drinking among Swedish ninth graders, and in 2017 a minority of Swedish ninth graders were alcohol consumers. There were small differences between drinkers and non-drinkers in self-rated health, psychosomatic problems, school situation and social position. This indicates a shift in the social marker of alcohol consumption among Swedish youth.

## Figures and Tables

**Table 1:** Comparisons of means between non-drinkers and drinkers.

	Non-Drinkers (*n* = 2987)		Drinkers (*n* = 1790)		HED (*n* = 355)	
	Mean	Std Dev	Mean	Std Dev	Mean	Std Dev
Self-rated health	**4.1**	**0.8**	**3.9**	**0.9**	3.9 *	1.0
PSP scale	**11.5**	**4.0**	**13.2**	**4.3**	13.7 *	*4.2*
SDQ internal	8.2	2.8	8.3	2.9	8.0	2.8
SDQ external	**7.3**	**2.0**	**8.1**	**2.2**	8.5 *	*2.4*
Grades	**43.0**	**11.2**	**42.0**	**11.0**	39.2 *	*12.6*
School satisfaction	**4.1**	**0.9**	**4.0**	**0.9**	3.9 *	1.0
Truancy	**1.3**	**0.8**	**1.7**	**1.2**	2.6 *	*1.7*
SDQ pro-social	**8.0**	**1.7**	**7.7**	**1.8**	7.4 *	*1.9*
Satisfied with friends	3.4	0.7	3.4	0.7	3.6 *	*0.6*

Bold text indicates a significant difference (*p* < 0.05) between non-drinkers and drinkers. Italics text indicates a significant difference (*p* < 0.05) between drinkers and HED. * indicates a significant difference (*p* < 0.05) between non-drinkers and HED.

**Table 2:** Differences between non-drinkers and drinkers by sex.

**Girls**	**Non-Drinkers (*n* = 1513)**		**Drinkers (*n* = 1152)**			**Boys**	**Non-Drinkers (*n* = 1474)**		**Drinkers (*n* = 1092)**		
	Mean	Std Dev	Mean	Std Dev	Ratio		Mean	Std Dev	Mean	Std Dev	Ratio
Self-rated health	**3.9**	**0.9**	**3.7**	**0.9**	1.05	Self-rated health	**4.3**	**0.7**	**4.2**	**0.8**	1.02
PSP scale	**12.7**	**4.1**	**14.7**	**4.0**	0.86	PSP scale	**10.2**	**3.5**	**11.7**	**4.1**	0.87
SDQ internal	**9.0**	**2.9**	**9.3**	**2.8**	0.97	SDQ internal	7.4	2.5	7.2	2.5	1.03
SDQ external	**7.3**	**2.0**	**8.1**	**2.1**	0.90	SDQ external	**7.3**	**2.1**	**8.2**	2.4	0.89
Grades	**44.4**	**11.1**	**43.0**	**10.7**	1.03	Grades	**41.6**	**11.2**	**40.0**	11.7	1.04
School satisfaction	**4.0**	**0.9**	**3.8**	**0.9**	1.05	School satisfaction	**4.2**	**0.8**	**4.1**	0.9	1.02
Truancy	**1.3**	**0.8**	**1.9**	**1.3**	0.68	Truancy	**1.3**	**0.8**	**1.8**	1.3	0.72
SDQ pro-social	**8.4**	**1.5**	**8.1**	**1.7**	1.04	SDQ pro-social	**7.6**	**1.8**	**7.3**	1.9	1.04
Satisfied with friends	3.4	0.7	3.4	0.7	1.00	Satisfied with friends	**3.4**	**0.7**	**3.5**	0.7	0.97

Bold text indicates significant (*p* < 0.05) differences between non-drinkers and drinkers.

## Data Availability

The data presented in this study are available on request from the corresponding author. The data are not publicly available due to privacy and ethical restrictions.

## References

[1] Rehm J., Mathers C., Popova S., Thavorncharoensap M., Teerawattananon Y., Patra J. (2009). Global burden of disease and injury and economic cost attributable to alcohol use and alcohol-use disorders. Lancet.

[2] Agardh E.E., Danielsson A.K., Ramstedt M., Ledgaard Holm A., Diderichsen F., Juel K., Vollset S.E., Knudsen A.K., Kinge J.M., White R. (2016). Alcohol-attributed disease burden in four Nordic countries: A comparison using the Global Burden of Disease, Injuries and Risk Factors 2013 study. Addiction.

[3] Leifman H., Kühlhorn E., Allebeck P., Andréasson S., Romelsjø A. (1995). Abstinence in late adolescence—Antecedents to and covariates of a sober lifestyle and its consequences. Soc. Sci. Med..

[4] Bailly D. (2016). The adolescent abstainers: A risk group?. L’encephale.

[5] Pedersen W. (2013). Abstainers: A risk group?. Tidsskr. Den Nor. Laegeforening Tidsskr. Prakt. Med. Ny Raekke.

[6] Mueller M., Kipke I., Frey F., Rossler W., Lupi G., Vetter S. (2009). Antecedents and covariates of alcohol consumption among Swiss male conscripts. Int. J. Environ. Res. Public Health.

[7] Raninen J., Leifman H., Ramstedt M. (2013). Who is not drinking less in Sweden? An analysis of the decline in consumption for the period 2004–2011. Alcohol Alcohol..

[8] Pape H. (1995). Om alkoholforskningens problemorientering [On the issue of problem orientation within the field of alcohol research]. Nord. Alkoholtisdkrift (Nord. Alcohol. Stud.).

[9] Norstrom T., Raninen J. (2015). Is there a link between per capita alcohol consumption and youth drinking? A time-series analysis for Sweden in 1972-2012. Addiction.

[10] Larm P., Åslund C., Raninen J., Nilsson K.W. (2018). Adolescent non-drinkers: Who are they? Social relations, school performance, lifestyle factors and health behaviours. Drug Alcohol. Rev..

[11] Hoel S., Magne Eriksen B., Breidablik H.J., Meland E. (2004). Adolescent alcohol use, psychological health, and social integration. Scand. J. Public Health.

[12] Lund I., Scheffels J. (2019). 15-year-old tobacco and alcohol abstainers in a drier generation: Characteristics and lifestyle factors in a Norwegian cross-sectional sample. Scand. J. Public Health.

[13] Skog O.J. (1985). The collectivity of drinking cultures: A theory of the distribution of alcohol consumption. Br. J. Addict..

[14] Demant J., Järvinen M. (2011). Social capital as norms and resources: Focus groups discussing alcohol. Addict. Res. Theory.

[15] Järvinen M., Room R. (2007). Youth Drinking Cultures: European Experiences.

[16] Jessor R. (1987). Problem-behavior theory, psychosocial development, and adolescent problem drinking. Br. J. Addict..

[17] Moffitt T.E. (1993). Adolescence-limited and life-course-persistent antisocial behavior: A developmental taxonomy. Psychol. Rev..

[18] Zetterqvist M. (2018). Skolelevers Drogvanor 2018.

[19] Vashishtha R., Pennay A., Dietze P., Marzan M.B., Room R., Livingston M. (2021). Trends in adolescent drinking across 39 high-income countries: Exploring the timing and magnitude of decline. Eur. J. Public Health.

[20] Pape H., Rossow I., Brunborg G.S. (2018). Adolescents drink less: How, who and why? A review of the recent research literature. Drug Alcohol Rev..

[21] Raitasalo K., Kraus L., Bye E.K., Karlsson P., Tigerstedt C., Törrönen J., Raninen J. (2021). Similar countries, similar factors? Studying the decline of heavy episodic drinking in adolescents in Finland, Norway and Sweden. Addiction.

[22] Raninen J., Livingston M. (2018). Exploring the changing landscape of youth drinking—We are still drawing the map. Drug Alcohol. Rev..

[23] Parker H.J., Parker H., Aldridge J., Measham F. (1998). Illegal Leisure: The Normalization of Adolescent Recreational Drug Use.

[24] Measham F., Newcombe R., Parker H. (1994). The normalization of recreational drug use amongst young people in north-west England. Br. J. Sociol..

[25] Goodman A., Lamping D.L., Ploubidis G.B. (2010). When to use broader internalising and externalising subscales instead of the hypothesised five subscales on the Strengths and Difficulties Questionnaire (SDQ): Data from British parents, teachers and children. J. Abnorm. Child Psychol..

[26] Vaughn M.G., Fu Q., Wernet S.J., DeLisi M., Beaver K.M., Perron B.E., Howard M.O. (2011). Characteristics of Abstainers from Substance Use and Antisocial in the United States. J. Crim. Justice.

[27] Scheffels J., Buvik K., Tokle R., Rossow I. (2020). Normalisation of non-drinking? 15–16-year-olds’ accounts of refraining from alcohol. Drug Alcohol. Rev..

[28] Törrönen J., Roumeliotis F., Samuelsson E., Kraus L., Room R. (2019). Why are young people drinking less than earlier? Identifying and specifying social mechanisms with a pragmatist approach. Int. J. Drug Policy.

[29] Sjödin L., Larm P., Karlsson P., Livingston M., Raninen J. (2021). Drinking motives and their associations with alcohol use among adolescents in Sweden. Nord. Stud. Alcohol Drugs.

[30] Vashishtha R., Livingston M., Pennay A., Dietze P., MacLean S., Holmes J., Herring R., Caluzzi G., Lubman D.I. (2020). Why is adolescent drinking declining? A systematic review and narrative synthesis. Addict. Res. Theory.

[31] Raninen J., Livingston M., Karlsson P., Leifman H., Guttormsson U., Svensson J., Larm P. (2018). One explanation to rule them all? Identifying sub-groups of non-drinking Swedish ninth graders. Drug Alcohol Rev..

